# Notch signaling: its roles and therapeutic potential in hematological malignancies

**DOI:** 10.18632/oncotarget.7772

**Published:** 2016-02-26

**Authors:** Yisu Gu, Massimo Masiero, Alison H. Banham

**Affiliations:** ^1^ Department of Clinical Haematology, Oxford University Hospitals, Churchill Hospital, Oxford, UK; ^2^ Nuffield Division of Clinical Laboratory Sciences, Radcliffe Department of Medicine, University of Oxford, Oxford, UK

**Keywords:** Notch, leukemia, lymphoma, hematology, therapy

## Abstract

Notch is a highly conserved signaling system that allows neighboring cells to communicate, thereby controlling their differentiation, proliferation and apoptosis, with the outcome of its activation being highly dependent on signal strength and cell type. As such, there is growing evidence that disturbances in physiological Notch signaling contribute to cancer development and growth through various mechanisms. Notch was first reported to contribute to tumorigenesis in the early 90s, through identification of the involvement of the *Notch1* gene in the chromosomal translocation t(7;9)(q34;q34.3), found in a small subset of T-cell acute lymphoblastic leukemia. Since then, Notch mutations and aberrant Notch signaling have been reported in numerous other precursor and mature hematological malignancies, of both myeloid and lymphoid origin, as well as many epithelial tumor types. Of note, Notch has been reported to have both oncogenic and tumor suppressor roles, dependent on the cancer cell type. In this review, we will first give a general description of the Notch signaling pathway, and its physiologic role in hematopoiesis. Next, we will review the role of aberrant Notch signaling in several hematological malignancies. Finally, we will discuss current and potential future therapeutic approaches targeting this pathway.

## INTRODUCTION

Notch is an evolutionally conserved signaling pathway consisting of a family of transmembrane receptors and ligands that allow cell-cell communication. Since its first discovery by Thomas Hunt Morgan in 1917 [1] in a mutant fly with notches, or ‘serration at the end of the wings’ [2], the role of Notch has been well characterized in the development of different tissues such as the processes of hematopoiesis and angiogenesis. This highly coordinated signaling system controls many aspects of cell biology, including differentiation, proliferation and death. Deregulation of the Notch pathway has been reported to play a role in the pathogenesis of a variety of solid tumors and this is summarized effectively by a number of recent reviews [3–6].

Importantly, the role of aberrant Notch signaling in hematological malignancies, which forms the focus of this review, has also emerged as an exciting field of cancer research. In fact, the most firmly established evidence for dysregulated Notch signaling in cancer is represented by the oncogenic *Notch1* receptor mutations present in over 50% of T-cell acute lymphoblastic leukemias (T-ALL) [7]. Interestingly, however, in another acute leukemia, acute myeloid leukemia (AML), Notch may act as a tumor suppressor [8, 9]. Also within mature B-cell neoplasms there is genetic evidence implicating the Notch pathway in disease pathogenesis, with *Notch1* receptor mutations representing an adverse prognostic marker in chronic lymphocytic leukemia (CLL) [10], and constitutive *Notch2* activation being reported in 8% of diffuse large B cell lymphomas (DLBCL) [11]. In this review we aim to introduce the Notch signaling pathway and summarize the evidence for its involvement in the pathogenesis and biology of hematological malignancies, together with the relevant therapeutic strategies currently in development.

## THE NOTCH SIGNALING PATHWAY

**Figure 1:**
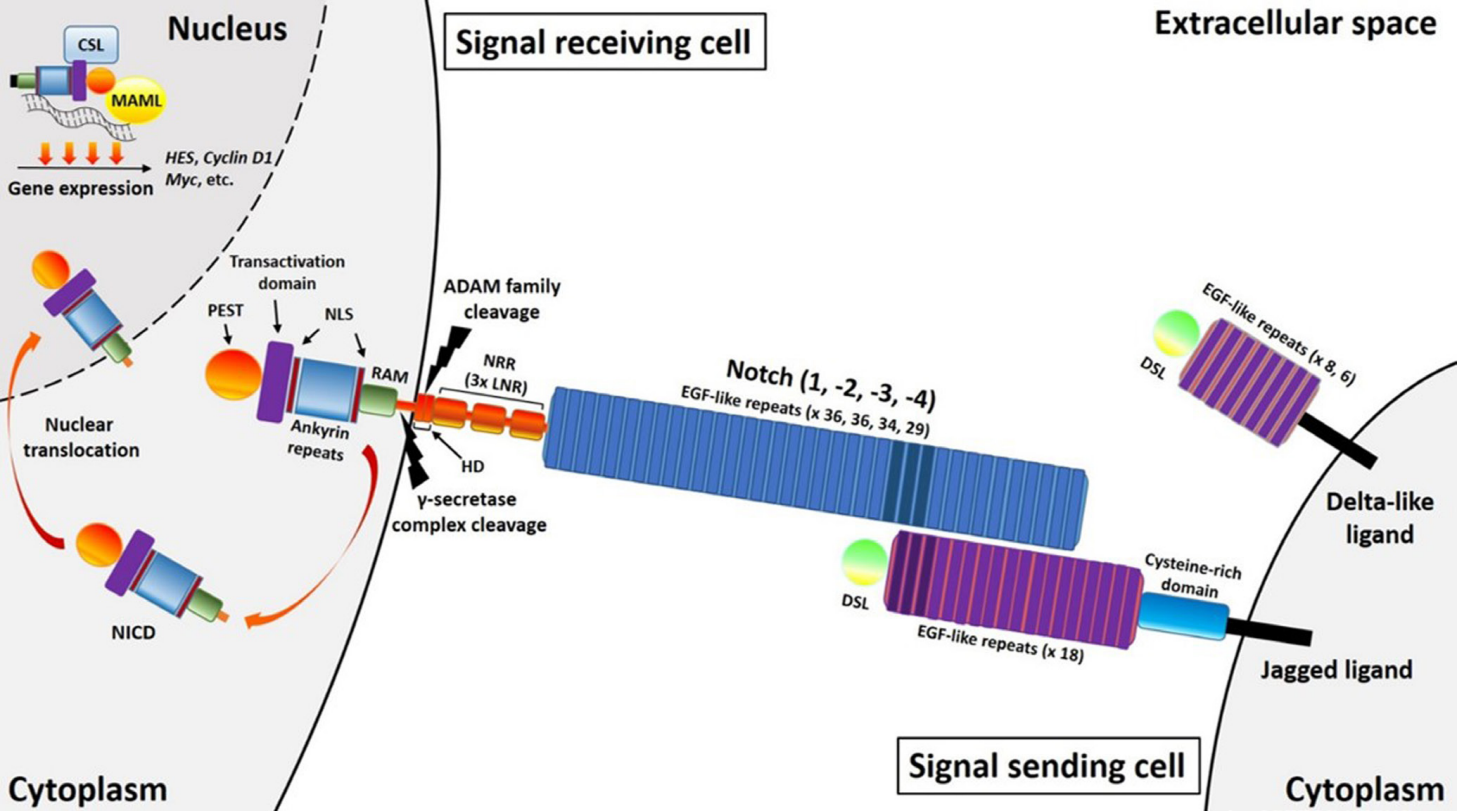
Brief overview of the Notch pathway Notch receptors have an extracellular domain comprising a variable number of epidermal growth factor (EGF)-like repeats (36 for Notch1-2, 34 for Notch3 and 29 for Notch4) and three Lin-12 Notch repeats (LNR). Ligand binding triggers sequential receptor cleavages involving ADAM family metalloproteases and the γ-secretase complex, ultimately leading to the cytoplasmic release of the intracellular domain (NICD). This comprises a RAM domain, six ankyrin repeats between two nuclear localization sequences (NLS) and a transactivation domain. A PEST sequence is also present at the C-terminus of all four Notch receptors, with a transactivation domain present in Notch1-2. After cytoplasmic release, this intracellular domain translocates into the nucleus where it exerts its transcriptional activity. Both Delta-like and Jagged ligands comprise of Delta-serrate-Lag2 (DSL) domains followed by a variable number of EGF-like repeats (8 for Delta-like1 and 4, and 6 for Delta-like3), with an additional cysteine-rich domain present in Jagged ligands. The Jagged ligands both have 18 EGF-like repeats.

First described in *Drosophila*, Notch comprises a receptor-ligand based signaling pathway that allows communication between neighboring cells. A varied combination of four transmembrane receptors (Notch1-4) and five transmembrane ligands (Delta-like1, 3 and 4, and Jagged1 and 2) are expressed on the surface of many different mammalian cell types. Central to this signaling pathway are the Notch receptors, whose largest portion is represented by the extracellular domain, which comprises a variable number of epidermal growth factor (EGF)-like repeats (36 for Notch1-2, 34 for Notch3 and 29 for Notch4) necessary for ligand-receptor interaction, and a negative regulatory region (NRR), composed of three Lin-12 Notch repeats (LNR), and a hetero-dimerization domain (HD) (Figure 1). The HD domain consists of an N-terminal and a C-terminal subunit, and in the absence of ligand stimulation, the LNR repeats fold over the HD domain to lock and stabilize the interaction of the two HD subunits. This LNR-HD unit maintains Notch in a resting state. However, upon ligand binding, the LNR repeats undergoes a conformational change, destabilizing the interaction between the two HD subunits and exposing the C-terminal subunit for cleavage. The intracellular domain of the Notch receptor (NICD) contains a PEST sequence at the C-terminus, a transactivation domain, 6 ankyrin repeats between two nuclear localization sequences (NLS) and a RAM domain. Both the Jagged and Delta-like ligands consist of a delta-serrate-Lag2 (DSL) domain at the N-terminus followed by 18 or 8 EGF-like repeats respectively, with the Jagged ligands also containing a cysteine-rich domain in the extracellular region. Following binding of the ligand DSL domain to the Notch receptor EGF-like repeats, the NICD is cleaved and released into the cytoplasm and then translocated into the nucleus. It is important to note the combinatorial complexity of this receptor-ligand binding, as a single receptor can be activated by multiple ligands, whilst one ligand can activate multiple receptors [12].

NICD release is a two-step process, with a first extracellular cleavage involving the ADAM family of metalloproteases, an α-secretase complex, followed by an intracellular step driven by a presenilin/γ-secretase protease, a complex consisting of presenilin, nicastrin, Pen-2 and Aph-1. Subsequent NICD translocation into the nucleus results in CSL (CBF1, Suppressor of hairless, and Lag-1) -dependent transcription activation. Here, an active Notch transcriptional complex is formed through the interaction between the NICD RAM domain with a CSL transcription factor, and a Mastermind-like (MAML) co-activator. *MAML* are a family of genes that encode critical transcriptional co-activators essential for Notch signaling. The three members of this family (MAML1-3), display different functions and expression patterns, increasing and modulating the diversity of signals deriving from Notch receptor-ligand binding in various cell types [13]. Among the primary targets of Notch signaling there are two families of transcriptional modulators: Hes (Hairy and E (spl)) and Hey/Hesr [14]. Both act as transcriptional repressors, inhibiting the expression of several genes. Other important targets of the Notch signaling pathway include: NF-κB [15], Cyclin D1 [16], p21 [17], GATA3 [18], c-Myc [19] and Deltex1[20].

There is also strong evidence for non-canonical, CSL-independent Notch signaling, of which there may be more than one pathway [21, 22]. For example, the NICD may interact with transcription factors not belonging to the CSL family, such as Lef1, HIF, and Mef2 [23–25]. Notch may also directly interact and modify the functions of cytoplasmic proteins, such as the translational regulator Musashi, without affecting its gene expression [21]. Other mechanisms of CSL-independent Notch signaling include the release of a different intracellular fragment to NICD by a protease distinct from presenilin, and the interaction of the cytoplasmic protein Deltex, with the ankyrin repeats of the Notch receptor [21].

Finally, mechanisms must be in place to switch off Notch signaling. One such process involves the mammalian Sel-10 homolog, an F-box protein (FBXW7), which is involved in ubiquitin-mediated protein degradation of the NICD *via* ubiquitination of its PEST domain [26–28]. There is also evidence that Notch signaling may be self-limiting, with MAML able to stimulate phosphorylation and proteolytic turnover of the NICD [29].

## THE ROLE OF NOTCH PATHWAY IN NORMAL HEMATOPOIESIS

Under physiological conditions, Notch plays a crucial role in the development of different tissues/organs [30]. Amongst these, it is pivotal in the generation of the embryonic hematopoietic stem cells (HSC) [31], in several stages of T-cell development [32–34] and in marginal zone B-cell development [35–37], while there is also a role for Notch signaling in myelopoiesis [38]. Furthermore, Notch signaling also plays an indirect role in HSC development, in addition to its direct role in HSC formation described below [39], as it is a key pathway in vascular development and arterial vessel identity, which in turn is significant in HSC development as these cells arise from the ventral wall of the dorsal aorta and vitelline and umbilical arteries [40, 41].

The hematopoietic system originates from several different sites during embryonic development. The most primitive HSC appear in the extraembryonic yolk sac, before migrating to intraembryonic sites, which includes the aorta-gonad mesonephros (AGM). Later, hematopoiesis switches to the fetal liver before its final transition to the bone marrow (BM). A number of studies highlight the essential role of Notch signaling for the development of definitive hematopoiesis in the embryo: Kumano *et al.* demonstrated the importance of Notch1, but not Notch2, in the generation of HSC from endothelial cells in embryonic hematopoiesis [42]. In addition, there is a critical role for Jagged1 mediated Notch1 activation, necessary for controlling GATA2 transcription factor expression within the AGM for maintenance of intraembryonic hematopoiesis independently of its role in arterial development in the mouse [43]. There is also evidence for the role of Notch in the development and maintenance of HSC in adult BM. In particular, parathyroid hormone (PTH) or PTH-related protein (PTHrP) receptor signaling stimulates osteoblastic cells, resulting in increased HSC numbers through Notch pathway [44].

There is also growing evidence for the role of NOTCH pathway exerting its influence through stromal cell interactions within the BM hematopoietic stem cell microenvironment. The latter is a highly specialized micro-anatomical compartment for the maintenance of HSC, allowing their differentiation into the full repertoire of peripheral blood cells. Within this environment, or ‘niche’, reside a number of mesenchymal and stromal cells that contribute towards hematopoiesis, with Notch signaling promoting proliferation, self-renewal and maintenance of HSC in the undifferentiated state [31, 45–48]. In addition, NOTCH signaling in the bone marrow microenvironment is thought to be necessary to maintain normal hematopoiesis. Within this microenvironment, osteoblastic cells are in close proximity to HSC, and may, through NOTCH signaling, provide a niche for their growth and development [44, 49].

Importantly, alongside this role, murine models have also proven that alterations in BM stromal cells can change Notch signaling from the microenvironment, inducing malignant outgrowth of pre-leukemic clones, indicating that appropriately regulated Notch signaling is key for proper hematopoietic development [50–53]. Therefore, developing a clearer understanding of the role of Notch pathway in the BM microenvironment control of stem cell activity will potentially support the identification of therapeutic targets.

Despite the important role in the BM microenvironment, the most extensively characterized function of Notch signaling in the hematopoietic system is in thymic T-cell development. BM HSC travel to the thymus *via* the bloodstream where they adopt a T-cell fate through successful β selection before differentiation through the double positive (CD4^+^CD8^+^) stage and subsequent maturation into single positive (CD4^+^ or CD8^+^) T cells upon TCRα rearrangement. Notch signaling is fundamental during early T-cell development, up to and including β selection. The earliest stages of this process involve the interaction between precursor cells' Notch1 with Delta-like ligand 4 (Dll4) expressed by thymic epithelial cells, with inactivation of the latter resulting in a complete block of T-cell development and concurrent ectopic B-cell formation [34]. Accordingly, constitutive Notch activation halts B-cell development and induces thymus-independent T-cell development [54].

Despite its suppressive role in early B-cell fate specification, Notch also plays a role in marginal zone B-cell (MZB) development in the spleen. In this organ there are two main B-cell subsets (the progenitors of which are derived from the BM) MZB and follicular B cells, the latter being the more abundant of the two subsets. MZB are able to rapidly respond to blood-borne antigens, such as those derived from pathogens, through the production of T-cell independent antibodies. B-cell development involves the rearrangement of heavy and light immunoglobulin chain genes to express a specific B-cell receptor (BCR), with cells expressing a self-reacting BCR undergoing clonal deletion. Further maturation in the spleen involves transitional T1 and T2 stages, before final differentiation into follicular B or MZB cells. In this context, interaction between B-cell expressed Notch2, and Delta-like ligand 1 (Dll1) expressed by both B and dendritic cells, is crucial for MZB cell fate specification, with the complete absence of MZB in *Dll1*-null mice [35]. In mature B cells, Notch signaling is mainly mediated by Notch2, with impaired pre-MZB and MZB cell differentiation in mice with heterozygous *Notch2* inactivation and total block of MZB differentiation before the pre-MZB stage on complete *Notch2* deletion [36].

## NOTCH IN PRECURSOR LYMPHOID AND MYELOID NEOPLASMS

**Figure 2:**
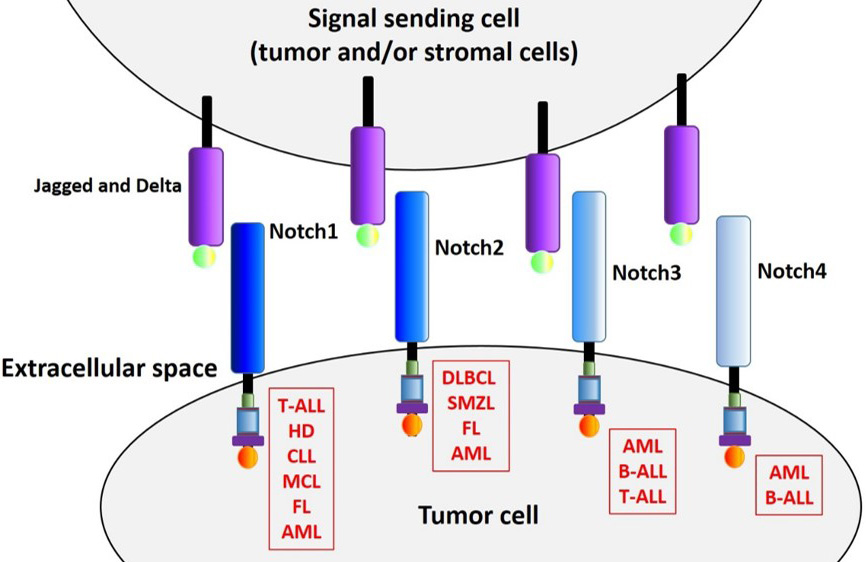
Notch receptors in hematological malignancies All four Notch receptors have been reported to play a role in multiple hematological malignancies. The mechanism of receptor activation and subsequent aberrant activity relies in some case on genetic mutations, often affecting the receptor itself (as in the case of *Notch1* in T-ALL), but also targeting signaling negative regulators such as *FBXW7*, an ubiquitin ligase that is implicated in NICD turnover. In some instances, increased pathway activation is due to receptor up-regulation or increased expression of specific ligands, the latter occurring in tumor cells and/or stromal cells (see text for more details).

Hematological malignancies encompass a wide range of pathologies and can broadly be divided into precursor (acute myeloid and lymphoblastic leukemias) and more mature subtypes (mature peripheral B/T-cell neoplasms, myeloma and Hodgkin lymphoma). Precursor malignancies typically exhibit aggressive disease progression, involving rapid proliferation of minimally differentiated cells. In comparison, mature neoplasms generally develop more slowly, and display a partial maturation phenotype [55]. We will now review the various mechanisms by which the Notch pathway contributes to the biology of several hematological malignancies (see also summary list in Table 1 and illustration in Figure 2).

**Table 1:** The role of Notch in individual cancer types

Tumor type	Oncogene or tumor suppressor	Mechanism	Reference
T-acute lymphoblastic leukemia	Oncogene	Constitutive activation of mutated Notch1 receptor results in aberrant continued signaling. Notch3 also has oncogenic function	[7, 58, 59, 81]
B-acute lymphoblastic leukemia	Oncogene	Notch3 and Notch4 mediate stromal cell-mediated anti-apoptotic effect on B-cells	[86, 160]
Acute myeloid leukemia	Tumor suppressor	Activation of all four Notch receptors induces apoptosis of AML cells	[8, 9]
Multiple myeloma	Oncogene	Homotypic and heterotypic interactions between plasma cells and BM stromal cells allow for anti-apoptosis through activation of Notch pathway	[89, 91, 161]
Hodgkin lymphoma	Oncogene	Constitutive activation of Notch1, possibly via NF-κB signaling pathway, promotes proliferation and survival in HRS cells	[94, 95, 162, 163]
Burkitt lymphoma	Oncogene	Notch signaling essential for Raji cell survival through maintenance of c-myc expression	[96]
Diffuse large B-cell lymphoma	Oncogene	Gain-of-function *Notch2* mutations result in PEST domain deletion	[11]
Chronic lymphocytic leukemia	Oncogene	*Notch1* PEST domain mutations result in continued Notch1 signaling	[10]
Mantle cell lymphoma	Oncogene	*Notch1* mutations prevents FBXW7-mediated NICD degradation	[108]
Splenic marginal zone lymphoma	Oncogene	*Notch2* PEST domain mutations NICD cause impaired degradation	[109, 110]
Follicular lymphoma	Oncogene	Constitutive activation of Notch signaling due to *Notch1* and *Notch2* mutations	[115, 116]

### T-acute lymphoblastic leukemia

The most firmly established oncogenic role for Notch signaling derives from its important function in T-ALL, which is characterized by the clonal proliferation of T-lineage progenitor cells, representing approximately 12 and 24% of all pediatric and adult ALL cases respectively [56, 57]. Mutations in the Notch signaling pathway are detectable in over 50% of all T-ALL patients, with the Notch1 receptor being recognized as the most common oncogene [7]. The involvement of *Notch1* was first identified in the chromosomal translocation t(7;9)(q34;q34.3), detectable in 1% of T-ALL [58]. This rearrangement truncates the *Notch1* gene and juxtaposes it next to the *TCRB* locus, resulting in the constitutive activation of an abnormal Notch1 receptor, with potent oncogenic activity [59]. Indeed, studies show that irradiated mice transplanted with HSC expressing a truncated *Notch1* receptor gene develop an immature form of T-cell leukemia with 50% incidence [60].

Aside from the rare t(7;9)(q34;q34.3) translocation, the majority of other *Notch1* activating mutations result in disruption of regulatory mechanisms that normally prevent the uncontrolled activation of this receptor, resulting in enhanced/prolonged or constitutive signaling. The most commonly occurring mutations are the HD class 1 mutations (45% of all T-ALL), consisting of single amino acid substitutions or small in-frame insertions and deletions within exon 26 and 27 that destabilize the interaction between the N- and C-terminal HD subunits [7]. This results in increased signaling through either ligand-independent or ligand hypersensitive activation of Notch1. Rare HD class 2 in-frame insertions in exon 27 result in a displacement of the processing site for ADAM cleavage to the outside the protective HD-LNR complex, allowing for constitutive, ligand-independent metalloprotease processing [7, 61]. Another similar mechanism of constitutive Notch1 signaling, known as juxta-membrane expansion (JME), present in 3% of T-ALL, is due to in-frame insertion mutations in exon 28 that displace the HD-LNR complex away from the cell membrane, inducing a conformational change that allows for increased ADAM cleavage [62]. A common group of mutations occurring in 15% of T-ALL involve nonsense or truncating mutations in exons encoding the PEST domain. These delete recognition sequences required for the proteasomal degradation of the NICD by the FBXW7-SCF ubiquitin ligase complex [7], and lead to aberrantly prolonged NICD signaling in the nucleus. Similar molecular outcomes also occur in response to mutations affecting arginine residues in FBXW7 (in about 15% of T-ALL) that are involved in substrate-recognition of the PEST domain of activated Notch1, resulting in impaired degradation of NICD [63, 64].

Interestingly, these Notch receptor and pathway regulator mutations are not all equivalent in terms of oncogenic potential. For example, *Notch1* PEST domain alterations are weak, and are only functional in the presence of Notch ligands [7], whereas the tumor-inducing potential of *Notch1* HD domain mutations is higher, relying on both ligand hypersensitivity and ligand independent activation mechanisms [61]. Finally, 25% of T-ALL patients will have a disease that is driven by a combination of the above mutations, with a cumulative oncogenic effect based on very high levels of Notch signaling. Examples include HD mutations existing in combination with truncations of the PEST domain, or dual mutations involving HD and *FBXW7* [7].

Studies have investigated the biological consequences of increased Notch signaling in T-ALL and have identified Notch1-ICD as a transcriptional activator of several genes involved in cell growth and metabolism, among which are potent oncogenes such as *MYC* [19, 65]. Interestingly, many of the genes controlled by Notch1 involved in cell growth and proliferation are also targets for MYC, thus generating an amplified gene network leading to uncontrolled cell growth [66]. Notch1 signaling also activates the PI3K-AKT-mTOR pathway that regulates glucose uptake and glycolysis and also sustains cell growth during T-cell development [67]. In T-ALL blast cells, this is mediated *via* HES1, a transcriptional repressor induced by Notch signaling, that is able to down-regulate the expression of the PI3K pathway negative regulator, *PTEN* [68]. Furthermore, Notch1 signaling also up-regulates insulin-like growth factor 1 receptor and interleukin 7 receptor α chain, molecules that are both located upstream of the P13K pathway [69, 70]. Conditional deletion of Rictor, an mTOR (mammalian target of rapamycin) interacting protein and essential component of the mTORC2 complex, has demonstrated that this complex enables Notch signaling to regulate AKT and NF-κB pathways, promoting thymic T-cell development and T-ALL progression [71]. Other oncogenic mechanisms induced by Notch1 signaling to support T-ALL cell growth and survival include promoting G1/S cell cycle transition [72], and the up-regulation of the IκB-kinase with the consequent increase in nuclear NF-κB and its transcriptional activity [73].

While their oncogenic role is clear, the prognostic value of *Notch* pathway mutations remains controversial. In fact, despite a number of studies showing patients with *Notch1* and/or *FBXW7* mutations having greater steroid sensitivity and reduced rates of minimal residual disease [74–76], some later trials failed to show that these correlated with an overall improved clinical outcome [77, 78].

The cell intrinsic mechanisms of Notch activation in T-ALL described above may also be influenced by the interactions of leukemic blasts with stromal cells (extrinsic activation), as suggested by the *in vitro* maintenance and long-term growth of human T-ALL cells when cultured with mouse stromal cells expressing the Dll1 ligand [79]. Similar ligand-induced Notch signaling has also been proved for Dll4 and Jagged1, in both cases involving Notch3 [80, 81], another receptor reported to play a role in T-ALL although not mutated [82, 83]. Furthermore, T-ALL blasts in contact with BM stromal cells are rescued from apoptosis *via* an IL-7 dependent mechanism [84], that involves ICAM-1 and LFA-1 adhesion molecules [85], that are modulated by Notch activation [86]. Better understanding of the role of stromal cells in T-ALL Notch activation would enable future therapies that target both intrinsic and extrinsic mechanisms supporting oncogenic Notch signaling in this disease.

### B-acute lymphoblastic leukemia

Despite the well-documented role of Notch signaling in T-ALL, much less is known about its function in B-cell acute lymphoblastic leukemia (B-ALL), the most common form of both childhood and adult acute leukemia. It is well understood that the interaction between BM mesenchymal stromal cells and the lymphoid precursors is vital for the regulation of proliferation, differentiation and survival of both normal and malignant B-precursor cells. In this context, Nwabo Kamdje *et al.* have shown that inhibition of both receptors (Notch3 and Notch4) or ligands (Jagged1, Jagged2 and Dll1) in an *in vitro* BM co-culture system results in B-ALL cell apoptosis, suggesting a potential important role for Notch pathway in mediating the anti-apoptotic effect of BM stromal cells for neoplastic B-cells in the progression of B-ALL [87].

### Acute myeloid leukemia

Interestingly, in the case of acute myeloid leukemia (AML) the role of the Notch pathway is not well understood, with different reports describe contrasting results. Early studies reported a possible tumor-suppressor function for Notch signaling in this acute leukemia [8, 9]. In these, analysis of primary samples demonstrated that this pathway is silenced in cytogenetically normal AML patients, with reactivation of Notch signaling inducing rapid cell cycle arrest and apoptosis of AML cells, both *in vivo* and *in vitro* [8]. In addition, combined genetic inactivation of the Notch pathway with deletion of the myeloid suppressor gene, *TET2*, results in an AML-like disease *in vivo*, further suggesting a possible anti-tumor role. In another study, Kannan *et al.* showed that despite Notch receptor expression in human AML cell lines, their activation and downstream target gene levels were low, suggesting the pathway lacked activity in these cell lines [9]. Importantly, AML cell growth arrest and caspase-dependent apoptosis could be induced through activation of each of the four Notch receptors or by the Notch target gene *HES1*. From a translational point of view, both studies therefore suggested the potential therapeutic use of Notch agonists in the treatment of AML.

Despite these early reports, recent research has identified a potentially oncogenic role for Notch signaling in the earliest phases of AML leukemogenesis through a microenvironment regulated process. Kode *et al.* demonstrated in a murine model that activated β-catenin in osteoblasts stimulated the expression of the Notch ligand Jagged1 by these cells, with subsequent activation of Notch signaling in the leukemia-initiating long-term repopulating HSC progenitors, without changes in other BM hematopoietic stem and progenitor cell populations. Jagged1 expression in osteoblasts was thus shown to contribute to AML development in murine models and was detectable in human samples, although it is currently unclear whether Jagged1 is required for leukemia maintenance, or only its initiation [50].

## THE ROLE OF NOTCH IN MATURE B-CELL NEOPLASMS

The mature B-cell neoplasms show some similarities to the stage of normal B-cell development they derive from. During cell differentiation, normal progenitor B cells within the BM undergo immunoglobulin VDJ gene rearrangement, differentiating into naïve B cells with mature surface immunoglobulins. Upon encountering antigen within the germinal center of secondary lymphoid organs, normal naïve B cells undergo differentiation, proliferate and mature into post-germinal center lymphocytes, which includes antibody-secreting long-lived plasma cells and memory cells. Most cases of mantle cell lymphoma (MCL), termed pre-germinal center neoplasms, are thought to correspond to naïve B cells [88]. Diffuse large B-cell lymphoma (DLBCL) (germinal center subtype), follicular lymphoma, Burkitt lymphoma and Hodgkin lymphoma are thought to develop from B cells undergoing maturation within germinal centers, whereas marginal zone lymphoma, multiple myeloma, activated B-cell-like DLBCL and chronic lymphocytic leukemia (CLL) are classified as post-germinal center neoplasms [55].

### Multiple myeloma

This malignancy is characterized by the abnormal proliferation of clonal plasma cells with the secretion of a monoclonal immunoglobulin [89]. Notch is a vital signaling pathway that contributes to the pro-neoplastic interaction between BM stromal cells and malignant plasma cells, which is observed in the development of multiple myeloma. Two major types of Notch activation occur in the process of neoplastic plasma cell deregulation within the BM microenvironment. Firstly, autonomous homotypic interactions occur between plasma cells due to their concurrent expression of Notch1, Notch2, and Notch3 receptors and Notch ligands, of which Jagged2 in particular is often over-expressed and correlates positively with disease stage [90]. Secondly, heterotypic interactions are mediated by ligand-expressing BM stromal cells and macrophages, which allow activation of Notch receptors on myeloma cells. Notch pathway activation protects myeloma cells from apoptosis, and leads to increased resistance to chemotherapy [91]. Indeed, the use of γ-secretase inhibitors (GSIs; small molecule inhibitors; see paragraph on the development of Notch-targeting therapies) to therapeutically block Notch signaling increased apoptosis *via* down-regulation of the Notch target gene *HES1* and the subsequent up-regulation of the pro-apoptotic protein Noxa. In addition, the concurrent use of GSIs enhanced the cytotoxic effects of doxorubicin and melphalan chemotherapy [91]. Jundt *et al.* have also described the tumor-promoting role of Notch in myeloma, showing that Notch1, Notch2 and Jagged1 are strongly expressed in myeloma cell lines, but are often missing in normal plasma cells, with Notch activation through both homotypic and heterotypic interactions causing increased tumor cell growth [92].

### Hodgkin lymphoma

Hodgkin lymphoma is typically characterized by scattered mono-nucleated Hodgkin and multinucleated Reed-Sternberg (HRS) cells, representing clonal tumor cell populations derived from germinal center B cells within an inflammatory infiltrate [93]. A key survival mechanism of HRS cells is based on the constitutive activation of NF-κB signaling, which controls lymphoma cell proliferation and survival [94]. In this context, it has been shown that Notch1 and its ligand Jagged1 are strongly expressed in HRS cells, with pathway activation induced by the latter accelerating tumor cell growth and inhibiting arsenic-induced apoptosis [95]. Interestingly, the specific cross-talk between NF-κB and Notch1 was recently highlighted using GSIs, with *in vitro* pharmacological Notch inhibition leading to a dose-dependent reduction in NF-κB transcriptional activity in HRS cells, as also observed in T-ALL [96].

### Burkitt lymphoma

Burkitt lymphoma is a highly aggressive non-Hodgkin B-cell lymphoma (NHL) that develops as a result of the translocation and deregulation of the *c-Myc* gene on chromosome 8. Notch signaling has been reported to have a growth-promoting role in Burkitt lymphoma using the Raji cell line as a model for this disease. He *et al.* showed that stimulation of these cells with soluble fragments of human DLL1 activated Notch signaling, inducing *HES1 via* a γ-secretase-dependent mechanism. This activation synergized with B-cell receptor signaling stimulating cell proliferation, while repressing apoptosis, and both effects were prevented by GSIs treatment. Importantly, as observed in T-ALL, Notch pathway inhibition strongly reduced c-Myc protein levels, indicating that Notch signaling might play a role in the high c-Myc activity typical of Burkitt lymphomas [97].

As for other tumor types, a role for microenvironment-induced Notch signaling has also been reported for Burkitt lymphomas. Cao *et al.* showed that vascular endothelial cells support the expansion of c-myc-driven mouse lymphoma cells with aggressive features, such as chemotherapy-resistance, augmented growth and extra-nodal invasion. This occurs *via* endothelial Jagged1-dependent activation of the Notch2 receptor in lymphoma cells. Importantly, more aggressive features were observed in Burkitt lymphoma cases showing high levels of vascular endothelial Jagged1 in primary human lymphoma tissues. In addition, the endothelial Jagged1 within tumor capillaries can confer enhanced chemo-resistance and invasiveness to indolent lymphoma cells in both humans and mice [98]. These findings may provide a useful means to stratify those patients presenting high Jagged1 levels in tumor-associated endothelial cells as high risk.

### Diffuse large B-cell lymphoma

DLBCL is a neoplasm of large B lymphoid cells and is the most common lymphoid malignancy in adults. Among the Notch receptors, Notch2 is preferentially expressed in mature B cells, and Lee *et al.* reported that 8% of DLBCL cases present with *Notch2* mutations, including nonsense mutations and single-base deletion, causing partial or complete deletion of the PEST domain, or one amino acid substitution in the protein's C-terminus. Some DLBCL cases also showed increased copy numbers of the mutated *Notch2* allele. As a consequence of PEST domain deletion, mutant cells were shown to have a ‘gain-of-function’ *Notch2* phenotype, with increased activity when stimulated by Notch ligands *in vitro*. It was however unclear from this study as to the prognostic significance and response to therapy outcomes of such mutations in DLBCL [11].

### Chronic lymphocytic leukemia

CLL is characterized by the progressive accumulation of mature, monomorphic B lymphocytes with high expression of CD23 and defective apoptosis, and it is the most common form of leukemia in adults over the age of 50 years [99]. Both receptors (Notch1 and Notch2) and ligands (Jagged1 and Jagged2) are expressed in CLL cells, leading to constitutively active Notch signaling that is not observed in normal B lymphocytes [100]. Importantly, genetic alterations are also reported, with *Notch1* PEST domain mutations being frequent in CLL and conferring an adverse prognosis. This was seen in a study of 209 patients, which found the *Notch1* PEST domain mutation to be mutually exclusive with genetic alterations in *TP53*, another poor prognostic marker in CLL. Both mutations were identified as independent predictors of poor survival outcome compared to wild-type genotypes. Further analysis from this study showed that those patients with *Notch1* mutations also presented with a more advanced stage of disease [10].

Of note, 80% of *Notch1* mutations in CLL involve c.7544_7545delCT, making it a potential target for molecular screening and future treatment strategies [101, 102]. Functionally, induction of Notch signaling *via* Jagged1 stimulation has been shown to increase the levels of both NF-κB activity and expression of cellular inhibitor of apoptosis protein 2 (c-IAP2) and X-linked inhibitor of apoptosis protein (XIAP) [100]. Conversely, GSIs and siRNA silencing of Notch2 increased B-CLL cell apoptosis through a reduction of NF-κB, c-IAP2 and XIAP. A number of other studies have also reported a higher frequency of *Notch1* mutations in cases of CLL undergoing Richter transformation to aggressive DLBCL, and in patients refractory to fludarabine therapy, further highlighting the potential clinical relevance of the Notch pathway in this disease [101, 103].

Several studies have shown the importance of BM mesenchymal stem cells in supporting the survival and preventing apoptosis of CLL cells [104–107]. These findings are consistent with an interesting clinical study using peripheral blood samples from patients with newly diagnosed CLL, and co-culture with human BM mesenchymal stromal cells of both autologous and allogeneic origin. This study found Notch1, Notch2, and Notch4 to be involved in stromal-dependent CLL resistance to fludarabine and cyclophosphamide chemotherapy, with mesenchymal stem cells dramatically increasing tumor cell survival. It is therefore thought that the mutations occurring in the *Notch1* gene may enhance the anti-apoptotic signals which the normal pathway already confers to CLL cells interacting with BM stromal cells [108].

### Mantle cell lymphoma

MCL represents a small subset of NHL, and is composed of monomorphic small to medium sized lymphoid cells involving the lymph nodes, spleen, blood and bone marrow. A study by Kridel *et al.* identified 12% of MCL harboring *Notch1* mutations, the majority consisting of a 2 base-pair deletion which truncates the C-terminal PEST domain, removing the recognition site for the FBXW7-SCF ubiquitin ligase degradation complex, resulting in a more stable and transcriptionally active form of Notch1-ICD [109]. Furthermore, this study observed that cases with *Notch1* mutations had a significantly shorter overall and progression-free survival, with inhibition of the Notch pathway resulting in increased apoptosis and reduced proliferation in two out of 10 MCL cell lines tested *in vitro*.

### Splenic marginal zone lymphoma

Splenic marginal zone lymphoma (SMZL) is a low-grade small B-cell lymphoma that involves the spleen and runs a relatively indolent course. *Notch* mutations have been shown in two studies to be the most frequent lesion, with *Notch2* gain-of-function gene mutations in approximately 20-25% of all SMZL cases, causing impaired degradation of the NICD in a similar fashion to *Notch1* mutations in MCL, CLL, and some forms of T-ALL [110, 111]. Notch2 signaling is needed for normal marginal zone B-cell development, and it is unclear whether *Notch2* mutations still require ligand binding for activation, or whether they are able to function through a ligand-independent signaling pathway. *Notch2* mutations show relative specificity for SMZL among B-cell lymphomas, being almost absent in other low grade malignancies of this family, and occurring infrequently in DLBCL [11]. Thus, specific *Notch2* mutations may potentially be used as a diagnostic marker to characterize this subset of indolent lymphoma. In addition, identification of *Notch2* mutations in SMZL opens an attractive opportunity for potential Notch-targeting therapies, with the appealing possibility of reducing the current use of splenectomy to control disease progression. Of note, the presence of *Notch2* mutations in SMZL has been reported by Kiel *et al.* to have adverse clinical outcomes in terms of disease relapse, histological transformation and patients' survival, and may therefore be used as biomarker for more aggressive therapeutic approaches [111]. Interestingly, similar *Notch2* germline mutations characterize the autosomal dominant bone disease Hajdu-Cheney syndrome, which however does not present with lymphoproliferative disorders, suggesting that additional mutations are necessary for B-cell transformation [112, 113].

### Follicular lymphoma

Follicular lymphoma (FL) is a common low-grade lymphoma, representing 20 to 25% of NHL cases in the US and Europe [114]. It is characterized by the chromosomal translocation t(14;18)(q32;q21), found in 80% of cases [115], and the role of Notch signaling in this disease is less established. Sanger sequencing in 114 cases of FL revealed a total of 6.1% with *Notch1* and/or *Notch2* mutations, all of which led to truncation of the PEST domain [116]. Interestingly, such mutations occurred exclusively in female patients, were more prevalent in cases lacking t(14;18)(q32;q21), and were significantly more frequent in those patients with splenic involvement and DLBCL transformation [117].

## NOTCH TARGETING IN STEM CELL TRANSPLANTATION

Beside its role in neoplastic growth, Notch signaling seems to play another important role for those patients with hematological malignancies requiring allogeneic hematopoietic stem cell transplantation (HSCT). This pathway has been shown to be involved in the pathogenesis of graft *versus* host disease (GVHD), which results from donor-derived T cells causing immune-mediated damage within host tissues and represents a key limitation for this therapeutic approach. A recent study showed that genetic and biochemical pan-blockade of Notch signaling within donor T cells in mouse models of allogeneic HSCT resulted in adequate protection from acute GVHD, including transplants with both major and minor histocompatibility antigen mismatch [118]. Notch inhibition reduced the production of inflammatory cytokines by donor T cells, such as IFNγ, TNFα, IL-17 and IL-2, whilst increasing production of regulatory T cells. Currently, treatment for acute GVHD involves strong immunosuppression to dampen the damaging effects of alloreactive T cells, but this unfortunately impairs the beneficial graft-*versus* leukemia (GVL) effect, with increased rates of disease relapse. Interestingly, T cells lacking Notch signaling retained their cytotoxic effects against allogeneic residual leukemic cells while sparing normal host cells. This dual advantage of reduced incidence of acute GVHD coupled with long-term disease-free survival post-transplant has made Notch signaling an attractive target for therapy [119]. A number of other studies have demonstrated similar results, with Zhang *et al.* reporting Notch-deprived donor T cells retaining their ability to engraft and proliferate in the host, but failing to accumulate in the gut and cause gastrointestinal acute GVHD [118].

## THE DEVELOPMENT OF NOTCH-TARGETING THERAPIES

The therapeutic rationale for the use of Notch-inhibiting strategies is based on the fact that blocking this pathway can have both direct effects on cancer cells, and can also indirectly hinder tumor growth by reducing blood supply through angiogenesis dysregulation and by disrupting the tumor stem cell niche [120, 121]. Furthermore, the fact that tumor cells often acquire multiple mutations in several different pathways interplaying with Notch, such as Hedgehog and WNT, supports the use of combination targeted therapies. Importantly, combination with traditional chemo-radiotherapy also seems promising as their therapeutic effect can also be enhanced by the use of Notch inhibitors [91] [122].

There are several Notch-targeting approaches currently in development, such as small molecules, namely γ- and α-secretase inhibitors (GSIs and ASIs respectively), immunotherapy with neutralizing antibodies targeting either Notch receptors or ligands, and the use of soluble recombinant ligands and receptors as decoys.

Most ongoing clinical trials testing Notch-targeting therapies in hematological malignancies, of which the majority are evaluating GSIs, involve patients with diseases that are refractory or relapsed to standard chemo-radiotherapy. There are currently few trials open and recruiting, but many more have been already terminated or withdrawn (Table 2). Current phase one clinical trials aim to explore the safety and maximum tolerable dose of anti-Notch therapies, either alone or in combination with standard therapies, with the goal of developing effective approaches that can reduce toxicities (see below), determine the most promising combination strategies, and identify the particular subsets of patients that are more likely to respond to Notch inhibition.

**Table 2:** Currently registered clinical trials targeting Notch pathway in hematological malignancies

	Intervention	Condition	Development Phase	Trial Status
NCT02518113	NICD inhibitor (LY3039478)	T-ALL	Phase1/ 2	Not yet recruiting
NCT01363817	γ-secretase inhibitor (BMS-906024)	T-ALL refractory to or relapsed from standard therapies	Phase 1	Open and currently recruiting
NCT01703572	Anti-Notch1 specific antibody (OMP-52M51)	Relapsed or refractory lymphoid malignancy	Phase 1	Open and currently recruiting
NCT01158404	Notch inhibitor (mechanism of action not stated)	Advanced solid tumor or lymphoma	Phase 1	Complete, no results available
NCT00878189	γ-secretase inhibitor (PF-03084014)	Advanced solid tumor malignancy and T-ALL	Phase 1	Terminated, no results available
NCT00100152	γ-secretase inhibitor (MK0752)	T-ALL	Phase 1	Terminated, no results available
NCT01088763	γ-secretase inhibitor (RO4929097)	Relapsed or refractory solid tumors, CNS tumors, lymphoma or T-ALL	Phase 1	Terminated, no results available
NCT01236586	γ-secretase inhibitor (RO4929097)	Pediatric relapsed/refractory solid or CNS tumors, lymphoma or T cell leukemia	Phase 1/ 2	Withdrawn, no results available
NCT01251172	γ-secretase inhibitor (RO4929097)	Multiple myeloma	Phase 2	Withdrawn, no results available

GSIs were originally developed for Alzheimer's treatment, due to the role played by the γ-secretase complex in processing the β-amyloid precursor protein in this disease [123]. They were quickly investigated for use in oncology and represent the first successful small molecule inhibitors of the Notch pathway. There have also been exciting results in the development of small molecules targeting the α-secretase complex, a multi-protein machinery responsible for the first of the two enzymatic cleavages releasing the NICD into the cytoplasm. GSIs are under extensive study for the treatment of both solid and hematological malignancies with T-ALL being one key target, due to the pivotal role played by Notch pathway in the disease. Pre-clinical studies show encouraging results with GSIs treatment of T-ALL cell lines resulting in the rapid clearance of activated Notch1 and the transcriptional down-regulation of its target genes, with consequent decrease in tumor cell growth and proliferation due to G1 cell cycle arrest [7, 59].

In addition to receptor processing blockade using small molecule inhibitors described above, highly specific antibodies targeting certain ligands, receptors and the γ-secretase complex have also been developed. GSIs main limitations reside in their ability to simultaneously inhibit all 4 Notch receptors and to target also other y-secretase regulated pathways. On the contrary, the superior specificity of antibody-based therapies avoid these limits by inhibiting only selected receptors or ligands, therefore sparing all others and preserving related desired Notch activity (e.g. in normal tissues). A number of different antibodies have been developed and tested for the treatment of both solid tumors and hematological malignancies. Notch1 is the most studied of the receptors, with a well described oncogenic function in several tissues and as such, it has seen the development of several neutralizing antibodies that have been pre-clinically tested for the treatment of T-ALL and several solid tumors, showing both a direct effect on tumor growth by inhibiting Notch pathway in neoplastic cells and an indirect effect by dysregulating angiogenesis [124–127]. Other receptors have also been successfully targeted including Notch2 and Notch3 [125, 128, 129]. It is important to note that when blocking Notch receptors, two main strategies have emerged in respect of the specific domain to be targeted. The first one, and perhaps the most intuitive one, aims at inhibiting the interaction between ligand and receptor by blocking the ligand-binding domain on the latter. This has proved to be an efficient approach when targeting wild type receptors, preventing ligand-dependent signaling, but failed when receptor mutations enabled ligand-independent pathway activation [124, 130]. As a consequence, antibodies capable of inhibiting mutated Notch activity have been generated, by targeting the receptor NRR. These are also able to prevent ligand-independent activation, as in the case of T-ALL [125, 127]. Among the ligands, inhibition of Dll4 has shown the most promising results, based on its effect on tumor angiogenesis and, in some case, on tumor cells directly (e.g. inhibition of cancer stem cells) [131–134]. Neutralizing antibodies against Jagged1 and Jagged1/2 have also been generated and shown to have therapeutic potential although they have not been characterized extensively [129, 135]. Lastly, anti-nicastrin monoclonal antibodies have also been developed and proven to have therapeutic potential in pre-clinical models of both T-ALL and breast cancer [136, 137]. Interestingly, the use of humanized monoclonal antibodies to block individual Notch receptors and ligands has also shown promising results in the HSCT setting. Tran *et al.* demonstrated that Notch1 and Delta-like ligands are key for mediating Notch signaling effects in alloreactive T cells during acute GVHD, with the specific blockade of Notch1 limiting GVHD but inducing intestinal side effects. However, inhibition of Dll1 or Dll4 prevented GVHD with few toxicities and preservation of the GVL effect [138].

Unfortunately, antibodies do face some limitations as a large proportion remains in the blood and their tumor uptake is dependent on the balance between their pharmacokinetics and the ability of the antibody to penetrate and be retained in the tissue. For example the large molecular weight of antibodies contributes to their long half-life by preventing renal clearance but can hamper their diffusion within solid tumors, while their high affinity enables tissue retention but may prevent effective tissue penetration. This is one of the reasons why antibody-based therapeutics have been particularly effective in hematological malignancies rather than solid tumors.

Another potential therapeutic approach involves the use of recombinant proteins or fragments acting as decoy molecules that inhibit Notch signaling. Examples include the use of soluble Jagged1 to inhibit pulmonary hypertension through its interference with ligand-induced signaling [139], the administration of Notch3-derived peptides to inhibit tumor growth in a pre-clinical model of lung cancer [140], and the use of EGF-like proteins DLK1 and DLK2 as inhibitory ligands of Notch1 [141]. Finally, the use of a Notch1 decoy was also shown to affect angiogenesis and reduce tumor growth *via* inhibition of Notch receptor activation [142]. A Mastermind inhibiting decoy peptide, has also been employed in a pre-clinical setting to block the interaction of the MAML protein with the NICD, resulting in direct inhibition of the Notch transcription factor complex [143].

Despite the great therapeutic potential however, limiting toxicities and on-target side effects associated with the various Notch therapies have been reported in both pre-clinical and clinical studies. For example, the success of GSI-based therapies in phase 1 trials has been hampered by their gastrointestinal toxicity, explained by normal Notch signaling directing gastrointestinal stem cells towards an epithelial fate, with subsequent blockade causing increased numbers of mucus-secreting goblet cells in the small intestine [144]. Similar health-threatening differentiation problems were also reported for Notch1 and Notch2 co-inhibition by monoclonal antibodies, although this was not investigated as a potential therapeutic combination [125]. Finally, another general concern stems from the complexity of the roles played by the Notch pathway, as it can play both oncogenic and tumor suppressive functions in a tissue-specific manner. The latter is the case of AML [8, 9], small cell lung cancer [145] and skin carcinomas [146], and there is therefore in theory the possibility that chronic suppression could increase the risk of this type of malignancies [145, 147]. An experimental proof for this comes from pre-clinical studies using Dll4-targeting antibodies, a therapeutic approach pursued by different biotech companies due to its powerful effect on tumor growth primarily *via* angiogenesis dysregulation [132, 133]. Genentech, one of these companies, reported concerning toxicities upon chronic treatment with their monoclonal antibody in pre-clinical models. These problems ranged from liver pathology to formation of vascular neoplasms due to abnormal endothelial cell activation, as normal Dll4 signaling is required to maintain these cells in a quiescent state [148].

A number of strategies have been trialed in an attempt to prevent or ameliorate the above side effects. The use of targeted approaches such as monoclonal antibodies already represents *per se* a safer option compared to current small molecule inhibitors, although not being completely risk-proof. Beside the Dll4-targeting issues previously mentioned, recent data have also shown that the therapeutic potential of double Jagged1/2 targeting is associated with important toxicities. A new technology however, was developed to make a combined Jagged1/2 neutralizing antibody active only in the tumor microenvironment (Probody technology), therefore sparing normal tissues while retaining the therapeutic effect [135]. Importantly, the development of safer approaches has not been based only on the identification of new drugs, and efforts have been made to optimize the use of effective but potentially toxic molecules. This is the case of GSIs, where the dose-limiting gut toxicity can be ameliorated by either intermittent dosing regimens, or concurrent administration of corticosteroids [149].

Other opportunities to selectively target Notch are provided by components of the downstream signaling pathway, such as individual Notch target genes. As several key Notch activated target genes are transcriptional regulators these could be targeted individually to partially block Notch signaling. For example HES1 has a crucial role in T-ALL and regulates a specific transcriptional signature that is similar to that modulated by the drug used to treat cardiac ischemia, perhexiline. While this compound inhibits mitochondrial carnitine palmitoyltransferase-1, and not HES1, perhexiline exhibited antitumor responses *in vivo* in a preclinical model of T-ALL and showed no significant adverse effects on the hematopoietic system [150]. HES1 silencing using overexpression of microRNA-199b-5p has also been shown to reduce engraftment of medulloblastoma by decreasing stem cell frequency, although additional target genes regulated by this microRNA may also contribute [151].

Finally, another area of opportunity for improvement resides in the possible synergistic effects of combining Notch therapy with inhibitors of other signaling pathways it interacts with, for example, Wnt [152], and NF-κB [153]. In particular, there have been examples of combined inhibition in solid tumors, such as with HER2 inhibitors in breast cancer [154, 155], with WNT-β-catenin in osteosarcoma [156], and with AKT in glioma [157]. Interestingly, within hematological malignancies, the frequent involvement of PI3K, mTOR and Notch pathways in T-ALL suggests a rationale base for the combined inhibition of all three pathways [158]. In addition, inhibition of Notch has also been reported to sensitize cancer cells to traditional chemotherapy in both solid tumors (such as the combined use of GSIs and the platinum based chemotherapy, oxaliplatin in colorectal cancer [159, 160], and combination with the nucleoside analogue, gemcitabine, for advanced solid tumors [161]), and hematological malignancies (GSIs significantly improved the cytotoxic effects of the chemotherapeutic drugs doxorubicin and melphalan in multiple myeloma [91]). However, a few studies have reported on the abrogation of chemotherapy-induced apoptosis when used in combination with GSIs in colon cancer [160] and T-ALL [162]. It is therefore vital to better understand the different mechanisms of action of Notch within different cancer cell types.

## CONCLUSIONS AND FUTURE DIRECTIONS

In this review, we have attempted to summarize the role of Notch pathway in hematological malignancies and the potential therapeutic approaches developed to date. Due to its important role in cancer stem cell biology, angiogenesis, cell proliferation and survival, Notch signaling represents one of the most promising molecular targets in cancer therapy, either alone or combination with traditional chemo-radiotherapy, and other targeted approaches. However, a few important hurdles still limit the direct translation of promising preclinical work into patient benefit, among which on-target toxicities and patient heterogeneity represent the main ones. To this end, we believe that future translational and clinical studies should therefore focus on the development of more selective therapeutic approaches (e.g. monoclonal antibodies), the discovery of biomarkers for patient selection (e.g. markers of sensitivity/resistance to Notch-targeting agents including ligand and receptor expression and activity as well as expression and mutational status of important pathway regulators), pharmacodynamics studies (to determine the most responsive Notch target genes and or generate antibodies to additional NICDs to enable correlation of the degree of pathway modulation with clinical outcomes), and on the identification of combination therapeutic approaches with enhanced efficacy and safety.

## References

[1] Morgan TH (1917). The Theory of the Gene. The American Naturalist.

[2] Dexter JS (1914). The analysis of a case of continuous variation in Drosophila by a study of its linkage relations. American Naturalist.

[3] Theys J, Yahyanejad S, Habets R, Span P, Dubois L, Paesmans K, Kattenbeld B, Cleutjens J, Groot AJ, Schuurbiers OC, Lambin P, Bussink J, Vooijs M (2013). High NOTCH activity induces radiation resistance in non small cell lung cancer. Radiother Oncol.

[4] Kanamori M, Kawaguchi T, Nigro JM, Feuerstein BG, Berger MS, Miele L, Pieper RO (2007). Contribution of Notch signaling activation to human glioblastoma multiforme. J Neurosurg.

[5] El Hindy N, Keyvani K, Pagenstecher A, Dammann P, Sandalcioglu IE, Sure U, Zhu Y (2013). Implications of Dll4-Notch signaling activation in primary glioblastoma multiforme. Neuro Oncol.

[6] Sun W, Gaykalova DA, Ochs MF, Mambo E, Arnaoutakis D, Liu Y, Loyo M, Agrawal N, Howard J, Li R, Ahn S, Fertig E, Sidransky D (2014). Activation of the NOTCH Pathway in Head and Neck Cancer. Cancer Res.

[7] Weng AP, Ferrando AA, Lee W, Morris JPt, Silverman LB, Sanchez-Irizarry C, Blacklow SC, Look AT, Aster JC (2004). Activating mutations of NOTCH1 in human T cell acute lymphoblastic leukemia. Science.

[8] Lobry C, Ntziachristos P, Ndiaye-Lobry D, Oh P, Cimmino L, Zhu N, Araldi E, Hu W, Freund J, Abdel-Wahab O, Ibrahim S, Skokos D, Armstrong SA (2013). Notch pathway activation targets AML-initiating cell homeostasis and differentiation. J Exp Med.

[9] Kannan S, Sutphin RM, Hall MG, Golfman LS, Fang W, Nolo RM, Akers LJ, Hammitt RA, McMurray JS, Kornblau SM, Melnick AM, Figueroa ME, Zweidler-McKay PA (2013). Notch activation inhibits AML growth and survival: a potential therapeutic approach. J Exp Med.

[10] Willander K, Dutta RK, Ungerback J, Gunnarsson R, Juliusson G, Fredrikson M, Linderholm M, Soderkvist P (2013). NOTCH1 mutations influence survival in chronic lymphocytic leukemia patients. BMC Cancer.

[11] Lee S-y, Kumano K, Nakazaki K, Sanada M, Matsumoto A, Yamamoto G, Nannya Y, Suzuki R, Ota S, Ota Y, Izutsu K, Sakata-Yanagimoto M, Hangaishi A (2009). Gain-of-function mutations and copy number increases of Notch2 in diffuse large B-cell lymphoma. Cancer Science.

[12] Fortini ME Notch Signaling: The Core Pathway and Its Posttranslational Regulation. Developmental Cell.

[13] Wu L, Sun T, Kobayashi K, Gao P, Griffin JD (2002). Identification of a family of mastermind-like transcriptional coactivators for mammalian notch receptors. Mol Cell Biol.

[14] Iso T, Kedes L, Hamamori Y (2003). HES and HERP families: multiple effectors of the Notch signaling pathway. J Cell Physiol.

[15] Oswald F, Liptay S, Adler G, Schmid RM (1998). NF-kappaB2 is a putative target gene of activated Notch-1 *via* RBP-Jkappa. Mol Cell Biol.

[16] Ronchini C, Capobianco AJ (2001). Induction of cyclin D1 transcription and CDK2 activity by Notch(ic): implication for cell cycle disruption in transformation by Notch(ic). Mol Cell Biol.

[17] Rangarajan A, Talora C, Okuyama R, Nicolas M, Mammucari C, Oh H, Aster JC, Krishna S, Metzger D, Chambon P, Miele L, Aguet M, Radtke F (2001). Notch signaling is a direct determinant of keratinocyte growth arrest and entry into differentiation. Embo j.

[18] Amsen D, Antov A, Jankovic D, Sher A, Radtke F, Souabni A, Busslinger M, McCright B, Gridley T, Flavell RA (2007). Direct regulation of Gata3 expression determines the T helper differentiation potential of Notch. Immunity.

[19] Weng AP, Millholland JM, Yashiro-Ohtani Y, Arcangeli ML, Lau A, Wai C, Del Bianco C, Rodriguez CG, Sai H, Tobias J, Li Y, Wolfe MS, Shachaf C (2006). c-Myc is an important direct target of Notch1 in T-cell acute lymphoblastic leukemia/lymphoma. Genes Dev.

[20] Izon DJ, Aster JC, He Y, Weng A, Karnell FG, Patriub V, Xu L, Bakkour S, Rodriguez C, Allman D, Pear WS (2002). Deltex1 redirects lymphoid progenitors to the B cell lineage by antagonizing Notch1. Immunity.

[21] Martinez Arias A, Zecchini V, Brennan K (2002). CSL-independent Notch signalling: a checkpoint in cell fate decisions during development?. Curr Opin Genet Dev.

[22] Andersen P, Uosaki H, Shenje LT, Kwon C (2012). Non-canonical Notch signaling: emerging role and mechanism. Trends Cell Biol.

[23] Gustafsson MV, Zheng X, Pereira T, Gradin K, Jin S, Lundkvist J, Ruas JL, Poellinger L, Lendahl U, Bondesson M (2005). Hypoxia requires notch signaling to maintain the undifferentiated cell state. Dev Cell.

[24] Ramain P, Khechumian K, Seugnet L, Arbogast N, Ackermann C, Heitzler P (2001). Novel Notch alleles reveal a Deltex-dependent pathway repressing neural fate. Curr Biol.

[25] Ross DA, Kadesch T (2004). Consequences of Notch-mediated induction of Jagged1. Exp Cell Res.

[26] Wu G, Lyapina S, Das I, Li J, Gurney M, Pauley A, Chui I, Deshaies RJ, Kitajewski J (2001). SEL-10 is an inhibitor of notch signaling that targets notch for ubiquitin-mediated protein degradation. Mol Cell Biol.

[27] Öberg C, Li J, Pauley A, Wolf E, Gurney M, Lendahl U (2001). The Notch Intracellular Domain Is Ubiquitinated and Negatively Regulated by the Mammalian Sel-10 Homolog. Journal of Biological Chemistry.

[28] Gupta-Rossi N, Le Bail O, Gonen H, Brou C, Logeat F, Six E, Ciechanover A, Israël A (2001). Functional Interaction between SEL-10, an F-box Protein, and the Nuclear Form of Activated Notch1 Receptor. Journal of Biological Chemistry.

[29] Fryer CJ, Lamar E, Turbachova I, Kintner C, Jones KA (2002). Mastermind mediates chromatin-specific transcription and turnover of the Notch enhancer complex. Genes Dev.

[30] Artavanis-Tsakonas S, Rand MD, Lake RJ (1999). Notch signaling: cell fate control and signal integration in development. Science.

[31] Pajcini KV, Speck NA, Pear WS (2011). Notch signaling in mammalian hematopoietic stem cells. Leukemia.

[32] Amsen D, Blander JM, Lee GR, Tanigaki K, Honjo T, Flavell RA (2004). Instruction of distinct CD4 T helper cell fates by different notch ligands on antigen-presenting cells. Cell.

[33] Koch U, Fiorini E, Benedito R, Besseyrias V, Schuster-Gossler K, Pierres M, Manley NR, Duarte A, Macdonald HR, Radtke F (2008). Delta-like 4 is the essential, nonredundant ligand for Notch1 during thymic T cell lineage commitment. J Exp Med.

[34] Hozumi K, Mailhos C, Negishi N, Hirano K, Yahata T, Ando K, Zuklys S, Hollander GA, Shima DT, Habu S (2008). Delta-like 4 is indispensable in thymic environment specific for T cell development. J Exp Med.

[35] Hozumi K, Negishi N, Suzuki D, Abe N, Sotomaru Y, Tamaoki N, Mailhos C, Ish-Horowicz D, Habu S, Owen MJ (2004). Delta-like 1 is necessary for the generation of marginal zone B cells but not T cells *in vivo*. Nat Immunol.

[36] Saito T, Chiba S, Ichikawa M, Kunisato A, Asai T, Shimizu K, Yamaguchi T, Yamamoto G, Seo S, Kumano K, Nakagami-Yamaguchi E, Hamada Y, Aizawa S (2003). Notch2 is preferentially expressed in mature B cells and indispensable for marginal zone B lineage development. Immunity.

[37] Tanigaki K, Han H, Yamamoto N, Tashiro K, Ikegawa M, Kuroda K, Suzuki A, Nakano T, Honjo T (2002). Notch-RBP-J signaling is involved in cell fate determination of marginal zone B cells. Nat Immunol.

[38] Schwanbeck R, Schroeder T, Henning K, Kohlhof H, Rieber N, Erfurth ML, Just U (2008). Notch signaling in embryonic and adult myelopoiesis. Cells Tissues Organs.

[39] Gering M, Patient R (2010). Notch signalling and haematopoietic stem cell formation during embryogenesis. J Cell Physiol.

[40] Bugeon L, Taylor HB, Progatzky F, Lin MI, Ellis CD, Welsh N, Smith E, Vargesson N, Gray C, Renshaw SA, Chico TJ, Zon LI, Lamb J (2011). The NOTCH pathway contributes to cell fate decision in myelopoiesis. Haematologica.

[41] de Bruijn MF, Speck NA, Peeters MC, Dzierzak E (2000). Definitive hematopoietic stem cells first develop within the major arterial regions of the mouse embryo. Embo j.

[42] Kumano K, Chiba S, Kunisato A, Sata M, Saito T, Nakagami-Yamaguchi E, Yamaguchi T, Masuda S, Shimizu K, Takahashi T, Ogawa S, Hamada Y, Hirai H (2003). Notch1 but not Notch2 is essential for generating hematopoietic stem cells from endothelial cells. Immunity.

[43] Robert-Moreno A, Guiu J, Ruiz-Herguido C, Lopez ME, Ingles-Esteve J, Riera L, Tipping A, Enver T, Dzierzak E, Gridley T, Espinosa L, Bigas A (2008). Impaired embryonic haematopoiesis yet normal arterial development in the absence of the Notch ligand Jagged1. Embo j.

[44] Calvi LM, Adams GB, Weibrecht KW, Weber JM, Olson DP, Knight MC, Martin RP, Schipani E, Divieti P, Bringhurst FR, Milner LA, Kronenberg HM, Scadden DT (2003). Osteoblastic cells regulate the haematopoietic stem cell niche. Nature.

[45] Carlesso N, Aster JC, Sklar J, Scadden DT (1999). Notch1-induced delay of human hematopoietic progenitor cell differentiation is associated with altered cell cycle kinetics. Blood.

[46] Kumano K, Chiba S, Shimizu K, Yamagata T, Hosoya N, Saito T, Takahashi T, Hamada Y, Hirai H (2001). Notch1 inhibits differentiation of hematopoietic cells by sustaining GATA-2 expression. Blood.

[47] Varnum-Finney B, Xu L, Brashem-Stein C, Nourigat C, Flowers D, Bakkour S, Pear WS, Bernstein ID (2000). Pluripotent, cytokine-dependent, hematopoietic stem cells are immortalized by constitutive Notch1 signaling. Nat Med.

[48] Stier S, Cheng T, Dombkowski D, Carlesso N, Scadden DT (2002). Notch1 activation increases hematopoietic stem cell self-renewal *in vivo* and favors lymphoid over myeloid lineage outcome. Blood.

[49] Li L, Milner LA, Deng Y, Iwata M, Banta A, Graf L, Marcovina S, Friedman C, Trask BJ, Hood L, Torok-Storb B (1998). The human homolog of rat Jagged1 expressed by marrow stroma inhibits differentiation of 32D cells through interaction with Notch1. Immunity.

[50] Kode A, Manavalan JS, Mosialou I, Bhagat G, Rathinam CV, Luo N, Khiabanian H, Lee A, Murty VV, Friedman R, Brum A, Park D, Galili N (2014). Leukaemogenesis induced by an activating [bgr]-catenin mutation in osteoblasts. Nature.

[51] Wang L, Zhang H, Rodriguez S, Cao L, Parish J, Mumaw C, Zollman A, Kamoka MM, Mu J, Chen DZ, Srour EF, Chitteti BR, HogenEsch H (2014). Notch-dependent repression of miR-155 in the bone marrow niche regulates hematopoiesis in an NF-kappaB-dependent manner. Cell Stem Cell.

[52] Kim YW, Koo BK, Jeong HW, Yoon MJ, Song R, Shin J, Jeong DC, Kim SH, Kong YY (2008). Defective Notch activation in microenvironment leads to myeloproliferative disease. Blood.

[53] Walkley CR, Olsen GH, Dworkin S, Fabb SA, Swann J, McArthur GA, Westmoreland SV, Chambon P, Scadden DT, Purton LE (2007). A microenvironment-induced myeloproliferative syndrome caused by retinoic acid receptor gamma deficiency. Cell.

[54] Pui JC, Allman D, Xu L, DeRocco S, Karnell FG, Bakkour S, Lee JY, Kadesch T, Hardy RR, Aster JC, Pear WS (1999). Notch1 expression in early lymphopoiesis influences B *versus* T lineage determination. Immunity.

[55] Swerdlow SH, Campo E, Harris N.L, Jaffe E.S, Pileri S.A, Stein H, Thiele J, Vardiman J.W (2008). WHO classification of tumours of haematopoietic and lymphoid tissues.

[56] Ferrando AA, Look AT (2000). Clinical implications of recurring chromosomal and associated molecular abnormalities in acute lymphoblastic leukemia. Semin Hematol.

[57] Pui CH, Relling MV, Downing JR (2004). Acute lymphoblastic leukemia. N Engl J Med.

[58] Ellisen LW, Bird J, West DC, Soreng AL, Reynolds TC, Smith SD, Sklar J (1991). TAN-1, the human homolog of the Drosophila notch gene, is broken by chromosomal translocations in T lymphoblastic neoplasms. Cell.

[59] Palomero T, Barnes KC, Real PJ, Glade Bender JL, Sulis ML, Murty VV, Colovai AI, Balbin M, Ferrando AA (2006). CUTLL1, a novel human T-cell lymphoma cell line with t(7;9) rearrangement, aberrant NOTCH1 activation and high sensitivity to gamma-secretase inhibitors. Leukemia.

[60] Pear WS, Aster JC, Scott ML, Hasserjian RP, Soffer B, Sklar J, Baltimore D (1996). Exclusive development of T cell neoplasms in mice transplanted with bone marrow expressing activated Notch alleles. J Exp Med.

[61] Malecki MJ, Sanchez-Irizarry C, Mitchell JL, Histen G, Xu ML, Aster JC, Blacklow SC (2006). Leukemia-associated mutations within the NOTCH1 heterodimerization domain fall into at least two distinct mechanistic classes. Mol Cell Biol.

[62] Sulis ML, Williams O, Palomero T, Tosello V, Pallikuppam S, Real PJ, Barnes K, Zuurbier L, Meijerink JP, Ferrando AA (2008). NOTCH1 extracellular juxtamembrane expansion mutations in T-ALL. Blood.

[63] Thompson BJ, Buonamici S, Sulis ML, Palomero T, Vilimas T, Basso G, Ferrando A, Aifantis I (2007). The SCFFBW7 ubiquitin ligase complex as a tumor suppressor in T cell leukemia. J Exp Med.

[64] O'Neil J, Grim J, Strack P, Rao S, Tibbitts D, Winter C, Hardwick J, Welcker M, Meijerink JP, Pieters R, Draetta G, Sears R, Clurman BE (2007). FBW7 mutations in leukemic cells mediate NOTCH pathway activation and resistance to gamma-secretase inhibitors. J Exp Med.

[65] Sharma VM, Calvo JA, Draheim KM, Cunningham LA, Hermance N, Beverly L, Krishnamoorthy V, Bhasin M, Capobianco AJ, Kelliher MA (2006). Notch1 contributes to mouse T-cell leukemia by directly inducing the expression of c-myc. Mol Cell Biol.

[66] Palomero T, Lim WK, Odom DT, Sulis ML, Real PJ, Margolin A, Barnes KC, O'Neil J, Neuberg D, Weng AP, Aster JC, Sigaux F, Soulier J (2006). NOTCH1 directly regulates c-MYC and activates a feed-forward-loop transcriptional network promoting leukemic cell growth. Proc Natl Acad Sci U S A.

[67] Chan SM, Weng AP, Tibshirani R, Aster JC, Utz PJ (2007). Notch signals positively regulate activity of the mTOR pathway in T-cell acute lymphoblastic leukemia. Blood.

[68] Palomero T, Sulis ML, Cortina M, Real PJ, Barnes K, Ciofani M, Caparros E, Buteau J, Brown K, Perkins SL, Bhagat G, Agarwal AM, Basso G (2007). Mutational loss of PTEN induces resistance to NOTCH1 inhibition in T-cell leukemia. Nat Med.

[69] Gonzalez-Garcia S, Garcia-Peydro M, Martin-Gayo E, Ballestar E, Esteller M, Bornstein R, de la Pompa JL, Ferrando AA, Toribio ML (2009). CSL-MAML-dependent Notch1 signaling controls T lineage-specific IL-7R{alpha} gene expression in early human thymopoiesis and leukemia. J Exp Med.

[70] Medyouf H, Gusscott S, Wang H, Tseng JC, Wai C, Nemirovsky O, Trumpp A, Pflumio F, Carboni J, Gottardis M, Pollak M, Kung AL, Aster JC (2011). High-level IGF1R expression is required for leukemia-initiating cell activity in T-ALL and is supported by Notch signaling. J Exp Med.

[71] Lee K, Nam KT, Cho SH, Gudapati P, Hwang Y, Park DS, Potter R, Chen J, Volanakis E, Boothby M (2012). Vital roles of mTOR complex 2 in Notch-driven thymocyte differentiation and leukemia. J Exp Med.

[72] Joshi I, Minter LM, Telfer J, Demarest RM, Capobianco AJ, Aster JC, Sicinski P, Fauq A, Golde TE, Osborne BA (2009). Notch signaling mediates G1/S cell-cycle progression in T cells *via* cyclin D3 and its dependent kinases. Blood.

[73] Shin HM, Minter LM, Cho OH, Gottipati S, Fauq AH, Golde TE, Sonenshein GE, Osborne BA (2006). Notch1 augments NF-kappaB activity by facilitating its nuclear retention. Embo j.

[74] Breit S, Stanulla M, Flohr T, Schrappe M, Ludwig WD, Tolle G, Happich M, Muckenthaler MU, Kulozik AE (2006). Activating NOTCH1 mutations predict favorable early treatment response and long-term outcome in childhood precursor T-cell lymphoblastic leukemia. Blood.

[75] Park MJ, Taki T, Oda M, Watanabe T, Yumura-Yagi K, Kobayashi R, Suzuki N, Hara J, Horibe K, Hayashi Y (2009). FBXW7 and NOTCH1 mutations in childhood T cell acute lymphoblastic leukaemia and T cell non-Hodgkin lymphoma. Br J Haematol.

[76] Asnafi V, Buzyn A, Le Noir S, Baleydier F, Simon A, Beldjord K, Reman O, Witz F, Fagot T, Tavernier E, Turlure P, Leguay T, Huguet F (2009). NOTCH1/FBXW7 mutation identifies a large subgroup with favorable outcome in adult T-cell acute lymphoblastic leukemia (T-ALL): a Group for Research on Adult Acute Lymphoblastic Leukemia (GRAALL) study. Blood.

[77] Mansour MR, Sulis ML, Duke V, Foroni L, Jenkinson S, Koo K, Allen CG, Gale RE, Buck G, Richards S, Paietta E, Rowe JM, Tallman MS (2009). Prognostic implications of NOTCH1 and FBXW7 mutations in adults with T-cell acute lymphoblastic leukemia treated on the MRC UKALLXII/ECOG E2993 protocol. J Clin Oncol.

[78] Zuurbier L, Homminga I, Calvert V, te Winkel ML, Buijs-Gladdines JG, Kooi C, Smits WK, Sonneveld E, Veerman AJ, Kamps WA, Horstmann M, Petricoin EF, Pieters R (2010). NOTCH1 and/or FBXW7 mutations predict for initial good prednisone response but not for improved outcome in pediatric T-cell acute lymphoblastic leukemia patients treated on DCOG or COALL protocols. Leukemia.

[79] Armstrong F, Brunet de la Grange P, Gerby B, Rouyez MC, Calvo J, Fontenay M, Boissel N, Dombret H, Baruchel A, Landman-Parker J, Romeo PH, Ballerini P, Pflumio F (2009). NOTCH is a key regulator of human T-cell acute leukemia initiating cell activity. Blood.

[80] Indraccolo S, Minuzzo S, Masiero M, Pusceddu I, Persano L, Moserle L, Reboldi A, Favaro E, Mecarozzi M, Di Mario G, Screpanti I, Ponzoni M, Doglioni C (2009). Cross-talk between tumor and endothelial cells involving the Notch3-Dll4 interaction marks escape from tumor dormancy. Cancer Res.

[81] Pelullo M, Quaranta R, Talora C, Checquolo S, Cialfi S, Felli MP, te Kronnie G, Borga C, Besharat ZM, Palermo R, Di Marcotullio L, Capobianco AJ, Gulino A (2014). Notch3/Jagged1 circuitry reinforces notch signaling and sustains T-ALL. Neoplasia.

[82] Bellavia D, Campese AF, Checquolo S, Balestri A, Biondi A, Cazzaniga G, Lendahl U, Fehling HJ, Hayday AC, Frati L, von Boehmer H, Gulino A, Screpanti I (2002). Combined expression of pTalpha and Notch3 in T cell leukemia identifies the requirement of preTCR for leukemogenesis. Proc Natl Acad Sci U S A.

[83] Masiero M, Minuzzo S, Pusceddu I, Moserle L, Persano L, Agnusdei V, Tosello V, Basso G, Amadori A, Indraccolo S (2011). Notch3-mediated regulation of MKP-1 levels promotes survival of T acute lymphoblastic leukemia cells. Leukemia.

[84] Barata JT, Cardoso AA, Boussiotis VA (2005). Interleukin-7 in T-cell acute lymphoblastic leukemia: an extrinsic factor supporting leukemogenesis?. Leuk Lymphoma.

[85] Winter SS, Sweatman JJ, Lawrence MB, Rhoades TH, Hart AL, Larson RS (2001). Enhanced T-lineage acute lymphoblastic leukaemia cell survival on bone marrow stroma requires involvement of LFA-1 and ICAM-1. Br J Haematol.

[86] Wang J, Fu L, Gu F, Ma Y (2011). Notch1 is involved in migration and invasion of human breast cancer cells. Oncol Rep.

[87] Nwabo Kamdje AH, Mosna F, Bifari F, Lisi V, Bassi G, Malpeli G, Ricciardi M, Perbellini O, Scupoli MT, Pizzolo G, Krampera M (2011). Notch-3 and Notch-4 signaling rescue from apoptosis human B-ALL cells in contact with human bone marrow-derived mesenchymal stromal cells. Blood.

[88] Hummel M, Tamaru J, Kalvelage B, Stein H (1994). Mantle cell (previously centrocytic) lymphomas express VH genes with no or very little somatic mutations like the physiologic cells of the follicle mantle. Blood.

[89] Bianchi G, Munshi NC (2015). Pathogenesis beyond the cancer clone(s) in multiple myeloma. Blood.

[90] Houde C, Li Y, Song L, Barton K, Zhang Q, Godwin J, Nand S, Toor A, Alkan S, Smadja NV, Avet-Loiseau H, Lima CS, Miele L (2004). Overexpression of the NOTCH ligand JAG2 in malignant plasma cells from multiple myeloma patients and cell lines. Blood.

[91] Nefedova Y, Sullivan DM, Bolick SC, Dalton WS, Gabrilovich DI (2008). Inhibition of Notch signaling induces apoptosis of myeloma cells and enhances sensitivity to chemotherapy. Blood.

[92] Jundt F, Probsting KS, Anagnostopoulos I, Muehlinghaus G, Chatterjee M, Mathas S, Bargou RC, Manz R, Stein H, Dorken B (2004). Jagged1-induced Notch signaling drives proliferation of multiple myeloma cells. Blood.

[93] Kuppers R (2002). Molecular biology of Hodgkin's lymphoma. Adv Cancer Res.

[94] Bargou RC, Emmerich F, Krappmann D, Bommert K, Mapara MY, Arnold W, Royer HD, Grinstein E, Greiner A, Scheidereit C, Dorken B (1997). Constitutive nuclear factor-kappaB-RelA activation is required for proliferation and survival of Hodgkin's disease tumor cells. J Clin Invest.

[95] Jundt F, Anagnostopoulos I, Forster R, Mathas S, Stein H, Dorken B (2002). Activated Notch1 signaling promotes tumor cell proliferation and survival in Hodgkin and anaplastic large cell lymphoma. Blood.

[96] Schwarzer R, Dorken B, Jundt F (2012). Notch is an essential upstream regulator of NF-kappaB and is relevant for survival of Hodgkin and Reed-Sternberg cells. Leukemia.

[97] He F, Wang L, Hu X-B, Yin D-D, Zhang P, Li G-H, Wang Y-C, Huang S-Y, Liang Y-M, Han H (2009). Notch and BCR signaling synergistically promote the proliferation of Raji B-lymphoma cells. Leukemia Research.

[98] Cao Z, Ding B-S, Guo P, Lee Sharrell B, Butler Jason M, Casey Stephanie C, Simons M, Tam W, Felsher Dean W, Shido K, Rafii A, Scandura Joseph M, Rafii S (2014). Angiocrine Factors Deployed by Tumor Vascular Niche Induce B Cell Lymphoma Invasiveness and Chemoresistance. Cancer Cell.

[99] Robak T, Jamroziak K, Robak P (2009). Current and emerging treatments for chronic lymphocytic leukaemia. Drugs.

[100] Rosati E, Sabatini R, Rampino G, Tabilio A, Di Ianni M, Fettucciari K, Bartoli A, Coaccioli S, Screpanti I, Marconi P (2009). Constitutively activated Notch signaling is involved in survival and apoptosis resistance of B-CLL cells. Blood.

[101] Fabbri G, Rasi S, Rossi D, Trifonov V, Khiabanian H, Ma J, Grunn A, Fangazio M, Capello D, Monti S, Cresta S, Gargiulo E, Forconi F (2011). Analysis of the chronic lymphocytic leukemia coding genome: role of NOTCH1 mutational activation. J Exp Med.

[102] Rossi D, Rasi S, Fabbri G, Spina V, Fangazio M, Forconi F, Marasca R, Laurenti L, Bruscaggin A, Cerri M, Monti S, Cresta S, Fama R (2012). Mutations of NOTCH1 are an independent predictor of survival in chronic lymphocytic leukemia. Blood.

[103] Puente XS, Pinyol M, Quesada V, Conde L, Ordonez GR, Villamor N, Escaramis G, Jares P, Bea S, Gonzalez-Diaz M, Bassaganyas L, Baumann T, Juan M (2011). Whole-genome sequencing identifies recurrent mutations in chronic lymphocytic leukaemia. Nature.

[104] Nishio M, Endo T, Tsukada N, Ohata J, Kitada S, Reed JC, Zvaifler NJ, Kipps TJ (2005). Nurselike cells express BAFF and APRIL, which can promote survival of chronic lymphocytic leukemia cells *via* a paracrine pathway distinct from that of SDF-1alpha. Blood.

[105] Burger JA, Tsukada N, Burger M, Zvaifler NJ, Dell'Aquila M, Kipps TJ (2000). Blood-derived nurse-like cells protect chronic lymphocytic leukemia B cells from spontaneous apoptosis through stromal cell-derived factor-1. Blood.

[106] Panayiotidis P, Jones D, Ganeshaguru K, Foroni L, Hoffbrand AV (1996). Human bone marrow stromal cells prevent apoptosis and support the survival of chronic lymphocytic leukaemia cells *in vitro*. Br J Haematol.

[107] Lagneaux L, Delforge A, Bron D, De Bruyn C, Stryckmans P (1998). Chronic lymphocytic leukemic B cells but not normal B cells are rescued from apoptosis by contact with normal bone marrow stromal cells. Blood.

[108] Nwabo Kamdje AH, Bassi G, Pacelli L, Malpeli G, Amati E, Nichele I, Pizzolo G, Krampera M (2012). Role of stromal cell-mediated Notch signaling in CLL resistance to chemotherapy. Blood Cancer J.

[109] Kridel R, Meissner B, Rogic S, Boyle M, Telenius A, Woolcock B, Gunawardana J, Jenkins C, Cochrane C, Ben-Neriah S, Tan K, Morin RD, Opat S (2012). Whole transcriptome sequencing reveals recurrent NOTCH1 mutations in mantle cell lymphoma. Blood.

[110] Rossi D, Trifonov V, Fangazio M, Bruscaggin A, Rasi S, Spina V, Monti S, Vaisitti T, Arruga F, Fama R, Ciardullo C, Greco M, Cresta S (2012). The coding genome of splenic marginal zone lymphoma: activation of NOTCH2 and other pathways regulating marginal zone development. J Exp Med.

[111] Kiel MJ, Velusamy T, Betz BL, Zhao L, Weigelin HG, Chiang MY, Huebner-Chan DR, Bailey NG, Yang DT, Bhagat G, Miranda RN, Bahler DW, Medeiros LJ (2012). Whole-genome sequencing identifies recurrent somatic NOTCH2 mutations in splenic marginal zone lymphoma. J Exp Med.

[112] Simpson MA, Irving MD, Asilmaz E, Gray MJ, Dafou D, Elmslie FV, Mansour S, Holder SE, Brain CE, Burton BK, Kim KH, Pauli RM, Aftimos S (2011). Mutations in NOTCH2 cause Hajdu-Cheney syndrome, a disorder of severe and progressive bone loss. Nat Genet.

[113] Brennan AM, Pauli RM (2001). Hajdu—Cheney syndrome: evolution of phenotype and clinical problems. Am J Med Genet.

[114] Salles GA (2007). Clinical Features, Prognosis and Treatment of Follicular Lymphoma. ASH Education Program Book.

[115] Yin CC, Luthra R (2013). Molecular detection of t(14;18)(q32;q21) in follicular lymphoma. Methods Mol Biol.

[116] Martinez D, Royo C, Castillo P, Valera A, Rosenwald A, Ott G, Esteban D, Gine E, Lopez-Guillermo A, Campo E (2013). Recurrent Mutations Of NOTCH Genes In Follicular Lymphoma. Blood.

[117] Karube K, Martinez D, Royo C, Navarro A, Pinyol M, Cazorla M, Castillo P, Valera A, Carrio A, Costa D, Colomer D, Rosenwald A, Ott G (2014). Recurrent mutations of NOTCH genes in follicular lymphoma identify a distinctive subset of tumours. J Pathol.

[118] Zhang Y, Sandy AR, Wang J, Radojcic V, Shan GT, Tran IT, Friedman A, Kato K, He S, Cui S, Hexner E, Frank DM, Emerson SG (2011). Notch signaling is a critical regulator of allogeneic CD4+ T-cell responses mediating graft-*versus*-host disease. Blood.

[119] Ebens CL, Maillard I (2013). Notch signaling in hematopoietic cell transplantation and T cell alloimmunity. Blood Reviews.

[120] Sharma A, Gadkari RA, Ramakanth SV, Padmanabhan K, Madhumathi DS, Devi L, Appaji L, Aster JC, Rangarajan A, Dighe RR (2015). A novel Monoclonal Antibody against Notch1 Targets Leukemia-associated Mutant Notch1 and Depletes Therapy Resistant Cancer Stem Cells in Solid Tumors. Sci Rep.

[121] Masuda S, Kumano K, Suzuki T, Tomita T, Iwatsubo T, Natsugari H, Tojo A, Shibutani M, Mitsumori K, Hanazono Y, Ogawa S, Kurokawa M, Chiba S (2009). Dual antitumor mechanisms of Notch signaling inhibitor in a T-cell acute lymphoblastic leukemia xenograft model. Cancer Sci.

[122] De Keersmaecker K, Lahortiga I, Mentens N, Folens C, Van Neste L, Bekaert S, Vandenberghe P, Odero MD, Marynen P, Cools J (2008). *In vitro* validation of gamma-secretase inhibitors alone or in combination with other anti-cancer drugs for the treatment of T-cell acute lymphoblastic leukemia. Haematologica.

[123] Evin G, Sernee MF, Masters CL (2006). Inhibition of gamma-secretase as a therapeutic intervention for Alzheimer's disease: prospects, limitations and strategies. CNS Drugs.

[124] Aste-Amezaga M, Zhang N, Lineberger JE, Arnold BA, Toner TJ, Gu M, Huang L, Vitelli S, Vo KT, Haytko P, Zhao JZ, Baleydier F, L'Heureux S (2010). Characterization of Notch1 antibodies that inhibit signaling of both normal and mutated Notch1 receptors. PLoS One.

[125] Wu Y, Cain-Hom C, Choy L, Hagenbeek TJ, de Leon GP, Chen Y, Finkle D, Venook R, Wu X, Ridgway J, Schahin-Reed D, Dow GJ, Shelton A (2010). Therapeutic antibody targeting of individual Notch receptors. Nature.

[126] Qiu M, Peng Q, Jiang I, Carroll C, Han G, Rymer I, Lippincott J, Zachwieja J, Gajiwala K, Kraynov E, Thibault S, Stone D, Gao Y (2013). Specific inhibition of Notch1 signaling enhances the antitumor efficacy of chemotherapy in triple negative breast cancer through reduction of cancer stem cells. Cancer Lett.

[127] Sharma A, Gadkari RA, Ramakanth SV, Padmanabhan K, Madhumathi DS, Devi L, Appaji L, Aster JC, Rangarajan A, Dighe RR (2015). A novel Monoclonal Antibody against Notch1 Targets Leukemia-associated Mutant Notch1 and Depletes Therapy Resistant Cancer Stem Cells in Solid Tumors. Scientific Reports.

[128] Li K, Li Y, Wu W, Gordon WR, Chang DW, Lu M, Scoggin S, Fu T, Vien L, Histen G, Zheng J, Martin-Hollister R, Duensing T (2008). Modulation of Notch signaling by antibodies specific for the extracellular negative regulatory region of NOTCH3. J Biol Chem.

[129] Huntzicker EG, Hotzel K, Choy L, Che L, Ross J, Pau G, Sharma N, Siebel CW, Chen X, French DM (2015). Differential effects of targeting Notch receptors in a mouse model of liver cancer. Hepatology.

[130] Yen WC, Fischer MM, Axelrod F, Bond C, Cain J, Cancilla B, Henner WR, Meisner R, Sato A, Shah J, Tang T, Wallace B, Wang M (2015). Targeting Notch signaling with a Notch2/Notch3 antagonist (tarextumab) inhibits tumor growth and decreases tumor-initiating cell frequency. Clin Cancer Res.

[131] Minuzzo S, Agnusdei V, Pusceddu I, Pinazza M, Moserle L, Masiero M, Rossi E, Crescenzi M, Hoey T, Ponzoni M, Amadori A, Indraccolo S (2015). DLL4 regulates NOTCH signaling and growth of T acute lymphoblastic leukemia cells in NOD/SCID mice. Carcinogenesis.

[132] Ridgway J, Zhang G, Wu Y, Stawicki S, Liang WC, Chanthery Y, Kowalski J, Watts RJ, Callahan C, Kasman I, Singh M, Chien M, Tan C (2006). Inhibition of Dll4 signalling inhibits tumour growth by deregulating angiogenesis. Nature.

[133] Noguera-Troise I, Daly C, Papadopoulos NJ, Coetzee S, Boland P, Gale NW, Lin HC, Yancopoulos GD, Thurston G (2006). Blockade of Dll4 inhibits tumour growth by promoting non-productive angiogenesis. Nature.

[134] Hoey T, Yen WC, Axelrod F, Basi J, Donigian L, Dylla S, Fitch-Bruhns M, Lazetic S, Park IK, Sato A, Satyal S, Wang X, Clarke MF (2009). DLL4 blockade inhibits tumor growth and reduces tumor-initiating cell frequency. Cell Stem Cell.

[135] Vasiljeva O, Menendez E, Sagert J, West JW, Richardson J, Desnoyers L, Liu S, Ford J, Plou K, Lowman H (2014). Abstract 2664: An anti-Jagged-1/−2 Probody demonstrates inhibition of Jagged-dependent Notch signaling and is activated in multiple types of tumors. Cancer Research.

[136] Hayashi I, Takatori S, Urano Y, Miyake Y, Takagi J, Sakata-Yanagimoto M, Iwanari H, Osawa S, Morohashi Y, Li T, Wong PC, Chiba S, Kodama T (2012). Neutralization of the gamma-secretase activity by monoclonal antibody against extracellular domain of nicastrin. Oncogene.

[137] Filipovic A, Lombardo Y, Faronato M, Abrahams J, Aboagye E, Nguyen QD, d'Aqua BB, Ridley A, Green A, Rahka E, Ellis I, Recchi C, Przulj N (2014). Anti-nicastrin monoclonal antibodies elicit pleiotropic anti-tumour pharmacological effects in invasive breast cancer cells. Breast Cancer Res Treat.

[138] Tran IT, Sandy AR, Carulli AJ, Ebens C, Chung J, Shan GT, Radojcic V, Friedman A, Gridley T, Shelton A, Reddy P, Samuelson LC, Yan M (2013). Blockade of individual Notch ligands and receptors controls graft-*versus*-host disease. J Clin Invest.

[139] Xiao Y, Gong D, Wang W (2013). Soluble JAGGED1 inhibits pulmonary hypertension by attenuating notch signaling. Arterioscler Thromb Vasc Biol.

[140] Lin L, Mernaugh R, Yi F, Blum D, Carbone DP, Dang TP (2010). Targeting specific regions of the Notch3 ligand-binding domain induces apoptosis and inhibits tumor growth in lung cancer. Cancer Res.

[141] Sanchez-Solana B, Nueda ML, Ruvira MD, Ruiz-Hidalgo MJ, Monsalve EM, Rivero S, Garcia-Ramirez JJ, Diaz-Guerra MJ, Baladron V, Laborda J (2011). The EGF-like proteins DLK1 and DLK2 function as inhibitory non-canonical ligands of NOTCH1 receptor that modulate each other's activities. Biochim Biophys Acta.

[142] Funahashi Y, Hernandez SL, Das I, Ahn A, Huang J, Vorontchikhina M, Sharma A, Kanamaru E, Borisenko V, Desilva DM, Suzuki A, Wang X, Shawber CJ (2008). A notch1 ectodomain construct inhibits endothelial notch signaling, tumor growth, and angiogenesis. Cancer Res.

[143] Moellering RE, Cornejo M, Davis TN, Del Bianco C, Aster JC, Blacklow SC, Kung AL, Gilliland DG, Verdine GL, Bradner JE (2009). Direct inhibition of the NOTCH transcription factor complex. Nature.

[144] Zecchini V, Domaschenz R, Winton D, Jones P (2005). Notch signaling regulates the differentiation of post-mitotic intestinal epithelial cells. Genes Dev.

[145] Sriuranpong V, Borges MW, Ravi RK, Arnold DR, Nelkin BD, Baylin SB, Ball DW (2001). Notch signaling induces cell cycle arrest in small cell lung cancer cells. Cancer Res.

[146] Lefort K, Mandinova A, Ostano P, Kolev V, Calpini V, Kolfschoten I, Devgan V, Lieb J, Raffoul W, Hohl D, Neel V, Garlick J, Chiorino G (2007). Notch1 is a p53 target gene involved in human keratinocyte tumor suppression through negative regulation of ROCK1/2 and MRCKalpha kinases. Genes Dev.

[147] Nicolas M, Wolfer A, Raj K, Kummer JA, Mill P, van Noort M, Hui CC, Clevers H, Dotto GP, Radtke F (2003). Notch1 functions as a tumor suppressor in mouse skin. Nat Genet.

[148] Yan M, Callahan CA, Beyer JC, Allamneni KP, Zhang G, Ridgway JB, Niessen K, Plowman GD (2010). Chronic DLL4 blockade induces vascular neoplasms. Nature.

[149] Real PJ, Tosello V, Palomero T, Castillo M, Hernando E, de Stanchina E, Sulis ML, Barnes K, Sawai C, Homminga I, Meijerink J, Aifantis I, Basso G (2009). Gamma-secretase inhibitors reverse glucocorticoid resistance in T cell acute lymphoblastic leukemia. Nat Med.

[150] Schnell SA, Ambesi-Impiombato A, Sanchez-Martin M, Belver L, Xu L, Qin Y, Kageyama R, Ferrando AA (2015). Therapeutic targeting of HES1 transcriptional programs in T-ALL. Blood.

[151] Garzia L, Andolfo I, Cusanelli E, Marino N, Petrosino G, De Martino D, Esposito V, Galeone A, Navas L, Esposito S, Gargiulo S, Fattet S, Donofrio V (2009). MicroRNA-199b-5p impairs cancer stem cells through negative regulation of HES1 in medulloblastoma. PLoS One.

[152] Espinosa L, Ingles-Esteve J, Aguilera C, Bigas A (2003). Phosphorylation by glycogen synthase kinase-3 beta down-regulates Notch activity, a link for Notch and Wnt pathways. J Biol Chem.

[153] Kuramoto T, Goto H, Mitsuhashi A, Tabata S, Ogawa H, Uehara H, Saijo A, Kakiuchi S, Maekawa Y, Yasutomo K, Hanibuchi M, Akiyama S, Sone S (2012). Dll4-Fc, an inhibitor of Dll4-notch signaling, suppresses liver metastasis of small cell lung cancer cells through the downregulation of the NF-kappaB activity. Mol Cancer Ther.

[154] Pandya K, Meeke K, Clementz AG, Rogowski A, Roberts J, Miele L, Albain KS, Osipo C (2011). Targeting both Notch and ErbB-2 signalling pathways is required for prevention of ErbB-2-positive breast tumour recurrence. Br J Cancer.

[155] Farnie G, Willan PM, Clarke RB, Bundred NJ (2013). Combined inhibition of ErbB1/2 and Notch receptors effectively targets breast ductal carcinoma *in situ* (DCIS) stem/progenitor cell activity regardless of ErbB2 status. PLoS One.

[156] Ma Y, Ren Y, Han EQ, Li H, Chen D, Jacobs JJ, Gitelis S, O'Keefe RJ, Konttinen YT, Yin G, Li TF (2013). Inhibition of the Wnt-beta-catenin and Notch signaling pathways sensitizes osteosarcoma cells to chemotherapy. Biochem Biophys Res Commun.

[157] Jin R, Nakada M, Teng L, Furuta T, Sabit H, Hayashi Y, Demuth T, Hirao A, Sato H, Zhao G, Hamada J (2013). Combination therapy using Notch and Akt inhibitors is effective for suppressing invasion but not proliferation in glioma cells. Neurosci Lett.

[158] Shepherd C, Banerjee L, Cheung CW, Mansour MR, Jenkinson S, Gale RE, Khwaja A (2013). PI3K/mTOR inhibition upregulates NOTCH-MYC signalling leading to an impaired cytotoxic response. Leukemia.

[159] Meng RD, Shelton CC, Li YM, Qin LX, Notterman D, Paty PB, Schwartz GK (2009). gamma-Secretase inhibitors abrogate oxaliplatin-induced activation of the Notch-1 signaling pathway in colon cancer cells resulting in enhanced chemosensitivity. Cancer Res.

[160] Timme CR, Gruidl M, Yeatman TJ (2013). Gamma-secretase inhibition attenuates oxaliplatin-induced apoptosis through increased Mcl-1 and/or Bcl-xL in human colon cancer cells. Apoptosis.

[161] Richter S, Bedard PL, Chen EX, Clarke BA, Tran B, Hotte SJ, Stathis A, Hirte HW, Razak AR, Reedijk M, Chen Z, Cohen B, Zhang WJ (2014). A phase I study of the oral gamma secretase inhibitor R04929097 in combination with gemcitabine in patients with advanced solid tumors (PHL-078/CTEP 8575). Invest New Drugs.

[162] Liu S, Breit S, Danckwardt S, Muckenthaler MU, Kulozik AE (2009). Downregulation of Notch signaling by gamma-secretase inhibition can abrogate chemotherapy-induced apoptosis in T-ALL cell lines. Ann Hematol.

